# Cognitive impairment based on computerized testing among patients with major depressive disorder after remission

**DOI:** 10.3389/fnhum.2026.1872083

**Published:** 2026-06-30

**Authors:** Cui Zhang, Fangfang Xu

**Affiliations:** Department of Geriatric Psychiatry, The Affiliated Brain Hospital of Nanjing Medical University, Nanjing, Jiangsu, China

**Keywords:** cognitive impairment, computerized testing, major depressive disorder, remission, risk factor

## Abstract

**Objectives:**

To investigate risk factors associated with cognitive impairment among patients achieving remission from major depressive disorder (MDD).

**Methods:**

This study was a retrospective analysis of data that had been prospectively collected from 1,227 patients diagnosed with MDD according to the Diagnostic and Statistical Manual of Mental Disorders (Fifth Edition). Remission was defined as achieving a total score of 7 or less on the 17-item Hamilton Rating Scale for Depression (HAMD-17) following treatment without the use of any antidepressants. Cognitive impairment was measured at 6 months after discharge using the computerized Testing with THINC-integrated tool. Patients’ demographic variables, clinical factors and self-reported rating scores were analyzed using multiple logistic regression model.

**Results:**

25.3% of patients exhibited cognitive impairment. Recurrent MDD (OR = 3.377, 95% CI: 1.629–7.003), increased inflammatory cytokines≥2 (OR = 4.032, 95% CI: 2.751–5.909), decreased dorsolateral prefrontal cortex volume (OR = 2.184, 95% CI: 1.298–3.676), decreased hippocampus volume (OR = 2.232, 95% CI: 1.393–3.576), increased amygdala volume (OR = 2.782, 95% CI: 1.751–4.420), decreased percentage of BOLD change for activity in subcortical network (OR = 2.689, 95% CI: 1.145–6.313) and ventral attention network (OR = 3.834, 95% CI: 1.573–9.345), increased percentage of BOLD change for activity in default mode network (OR = 5.136, 95% CI: 2.198–12.005) and Insomnia Severity Index scores ≥22 points (OR = 2.679, 95% CI: 1.502–4.779) were independent risk factors for cognitive impairment in remission from MDD. Receiver-operating characteristic curve demonstrated good predictive performance with area under the curve of 0.902.

**Conclusion:**

Our results identified factors associated with cognitive impairment in patients with MDD after remission, enabling improved monitoring and management for reducing these risk factors.

## Introduction

Major depressive disorder (MDD) is a mental disorder characterized by a persistent sad mood or interest loss in activities lasting for at least 2 weeks (Bains and Abdijadid, 2025). Between 1991 and 2021, the age-standardized prevalence of MDD in China increases from 1678.76 per 100,000 people to 1426.49 (Wang et al., 2025). The World Health Organization has projected that MDD will become the leading cause of disease burden globally by 2030 (Malhi and Mann, 2018). According to the Research Domain Criteria, cognitive impairment is one of the most common symptoms of MDD, including distorted information processing, heightened attention to negative stimuli, and impairments in attention, short-term memory and executive functioning (Knight and Baune, 2018). Research into the pathomechanisms indicates that immune system activates neurobiological mechanisms to release inflammatory mediators, which subsequently cause neuroinflammation. This leads to changes in volume, activity and connectivity of several cerebral regions involved in the production of cognitive deficits (Czerwinska and Pawlowski, 2020). Patients with MDD typically suffer from cognitive impairments both before and during acute episodes. However, most antidepressants cannot fully reverse deficits in cognitive function. Consequently, cognitive deficits often persists as residual symptoms, involving difficulties with aspects of cognitive function after the core symptoms of MDD are normalized, which in turn negatively affects long-term psychosocial function and quality of life (Lam et al., 2014). Current evidence suggests that targeted treatments such as psychological and pharmacological therapy are effective in addressing cognitive impairment in individuals with MDD (Douglas et al., 2020). From a precognitive perspective, clinicians are encouraged to routinely assess cognitive impairments and recognize the clinical factors associated with these symptoms in MDD patients to facilitate targeted interventions (Pan et al., 2017). The Thinking Health Integrated Neurocognitive Collection‌-Integrated Tool (THINC-it) is a validated computerized cognitive assessment battery designed as a quick, easy-to-use and practical screening tool. It evaluates both objective and subjective cognitive impairments in MDD, significantly shortening the administration time to less than 20 min compared to traditional neurophysiological assessments (Zhu et al., 2025).

With the assistance of THINC-it, this study was the first to investigate the characteristics and influencing factors of cognitive impairment following the remission of depressive symptoms in order to facilitate identification and targeted intervention in adults with MDD.

## Methods

### Study design and participant

The retrospective observational study was approved by the Ethics Examining Committee of the Affiliated Brain Hospital of Nanjing Medical University (NBH-clinical-2026013) in accordance with the Declaration of Helsinki. Results were reported following the Strengthening the Reporting of Observational Studies in Epidemiology (STROBE) guidelines (Vandenbroucke et al., 2014). Written informed consent for data publication was obtained from all patients.

Between January 1, 2023, and December 31, 2025, records of patients with MDD were examined using the electronic medical records (EMRs). Inclusion criteria were as follows: (1) a diagnosis of MDD according to the fifth edition of Diagnostic and Statistical Manual of Mental Disorders (DSM-5) (First, 2013); (2) achieving remission, defined as having a total score of 7 or less on the 17-item Hamilton Rating Scale for Depression (HAMD-17) after treatment without the use of any antidepressants (Nierenberg and DeCecco, 2001); (3) being free of antidepressants; (4) aged 18 years or older; (5) having complete medical records. Patients were excluded if they had bipolar disorder or schizophrenia; substance use disorder(Miguel et al., 2023; Hashemzadeh et al., 2021); primary neurocognitive disorder; major neurologic disorders affecting cognitive function such as cerebral palsy, Parkinson’s disease, stroke, brain trauma and epilepsy; and pregnancy or lactation.

### Outcomes measurement

We retrieved baseline of patients’ demographic characteristics from the medical records: age, gender, body mass index, race, education/martial/socioeconomic status, smoking status, physical activity. In addition, we extracted the clinical data from the EMRs: family depression history, first depressive episode duration, episode number, treatment-resistance, hospitalization number, suicide ideation frequency, current MDD duration, depression severity, treatment setting/type, antidepressant type/exposure time/ washout time, inflammatory cytokines, brain volumes, neural network activity, psychiatric/physical comorbidities, adjuvant psychotherapy, readmission status, sleep quality and life quality.

Inflammatory cytokines, such as C-reactive protein (CRP), interleukin-6 (IL-6) and tumor necrosis factor-*α* (TNF-α) were examined by the Enzyme-linked immunosorbent assay. The normal serum levels for CPR, IL-6 and TNF-α were ≤3 mg/L, ≤7 ρg/ml and ≤8 ρg/ml, respectively. The inflammatory burden was assessed based on patients’ respective inflammatory response, determined by the number of elevated inflammatory cytokines. Brain volumes were acquired using magnetic resonance imaging (MRI) with a 3 Tesla scanner, and were normalized for intracranial volume. Images were obtained using the magnetization-prepared rapid acquisition gradient echo sequence with a repletion time (TR) of 2,530 ms, an echo time (TE) of 1.85 ms, a flip angle (FA) of 15°, a matrix of 256 × 256, a field of view (FOV) of 256 × 256 mm^2^ and a voxel size of 1 × 1 × 1 mm^3^. Blood oxygen level dependent (BOLD) images were obtained during the monetary incentive delay (MID) task using functional magnetic resonance imaging (fMRI). Images were acquired using a gradient-echo echo-planner imaging sequence with a TR of 2000 ms, a TE of 30 ms, a FA = 90°, a matrix of 64 × 64, FOV of 200 × 200 mm^2^ and a voxel size of 3.13 × 3.13 × 4.2 mm^3^. Regions of interest were constructed for the default mode network (DMN), subcortical network (SCN) and ventral attention network (VAN) based on the human Brainnetome Atlas,^1^ which provides detailed anatomical and connectivity patterns for 210 cortical and 36 subcortical regions (Zhang et al., 2023). The percentage of BOLD signal change was calculated via the following formula: (S task-S baseline)/S baseline (Kundu et al., 2012). Two medical imaging specialists conducted the imaging analysis blindly, and data processing was performed using the fMRI PREProcessing workflows, version 25.2.4. At 6 months post-discharge, a brief cognitive function assessment was performed using the Chinese version of the THINC-it tool via a mobile application during an inpatient ward follow-up visit (Hou et al., 2020). The THINC-it tool test was administrated in the following sequence: one subjective test, the Perceived Deficits Questionnaire-5-item (PDQ-5-D), followed by four objective tests: the Spotter (SPO), Symbol Check (SC), Code Breaker (CB) and Trails (TRA). The PDQ-5-D consisted of five questions to measure the attention/concentration, planning/organization and both retrospective and prospective memory. Patients were asked to choose the response that best reflected their experience for over the previous 7 days. The SPO test assessed the attention/alertness and motor speed using a series of arrows, pointing either left or right. Patients had to quickly select the direction corresponding to the arrow. SC evaluated attention/alertness, working memory and motor speed with a sequence of laterally moving symbols. Patients were required to accurately recall the hidden symbols as quickly as possible. The CB test estimated attention/alertness and processing speed using six consecutively numbered symbols. Patients were asked to rapidly match each number to its corresponding symbol. The TRA test measured executive function by requiring patients to connect Chinese characters (壹to玖) and numbers (1 to 9) in the correct order. The composite THINC-it score integrated the total score from five cognitive tests. Patients were classified as having cognitive impairment if their cognitive performance was at least 1.0 standard deviation (SD) below the standardized mean scores of 100 healthy controls. These controls, matched to the MDD patients based on age, sex and education level, were not taking any medications that might affect cognitive function (McIntyre et al., 2017).

Severity of depressive symptoms was measured using the 17-item Hamilton Depression Rating Scale (HAMD-17). Higher total scores indicated more severe symptom (Nierenberg and DeCecco, 2001). Insomnia severity was assessed by the self-reported Insomnia Severity Index (ISI) with seven items. Responses were rated on a 5-piont Likert scale, yielding a total score ranging from 0 to 28. A total score of ≥22 was classified as severe insomnia (Bastien et al., 2001). Quality of life was evaluated via the World Health Organization Quality of Life Brief version (WHOQOL-BREF), which included physical, psychological, social and environmental domains. Higher scores indicated better QoL (Casamali et al., 2019).

### Sample size calculation

Sample size was calculated using PASS software, version 22 (NCSS, LLC., Kaysville, Utah, USA). This study aimed to investigate the relationship between risk factors and cognitive impairment after remission of MDD. According to prior evidence, the probability of cognitive impairment was reported to range from 22 to 30% among MDD remitters (Cokmus et al., 2021). Researchers wanted a sample size sufficient to detect an odds ratio (OR) of at least 1.50, considering the percentage of the independent variable of interest ranging from 1 to 50 by 1. To attain a 90% power with a two-sided test at the 0.05 significance level, a total of 1,136 patients were needed. After adjusting for an expected 20% data loss, the final sample included 1,420 cases.

### Statistical analysis

Statistics were performed using SPSS software, version 22.0 (SPSS Inc., Chicago, IL). A two-sided *p* < 0.05 was considered statistically significant. Kolmogorov–Smirnov Z test was utilized to assess data normality. Normally distributed continuous data and categorical data were presented as mean ± SD and percentage, and Student t test and Chi-square test were employed for comparison, respectively. Variance inflation factor (VIF) was used for multicollinearity between variables, with a VIF ≥ 10 indicating significant problem of collinearity. Multiple logistic regression using likelihood ratio stepwise model was performed to identify risk factors that predicted cognitive impairment among patients with MDD after remission. A prognostic index (PI) was calculated based on the regression coefficients. Receiver-operating characteristic curves (ROC) were plotted to test the performance of the predictive model.

## Results

**Figure 1:**
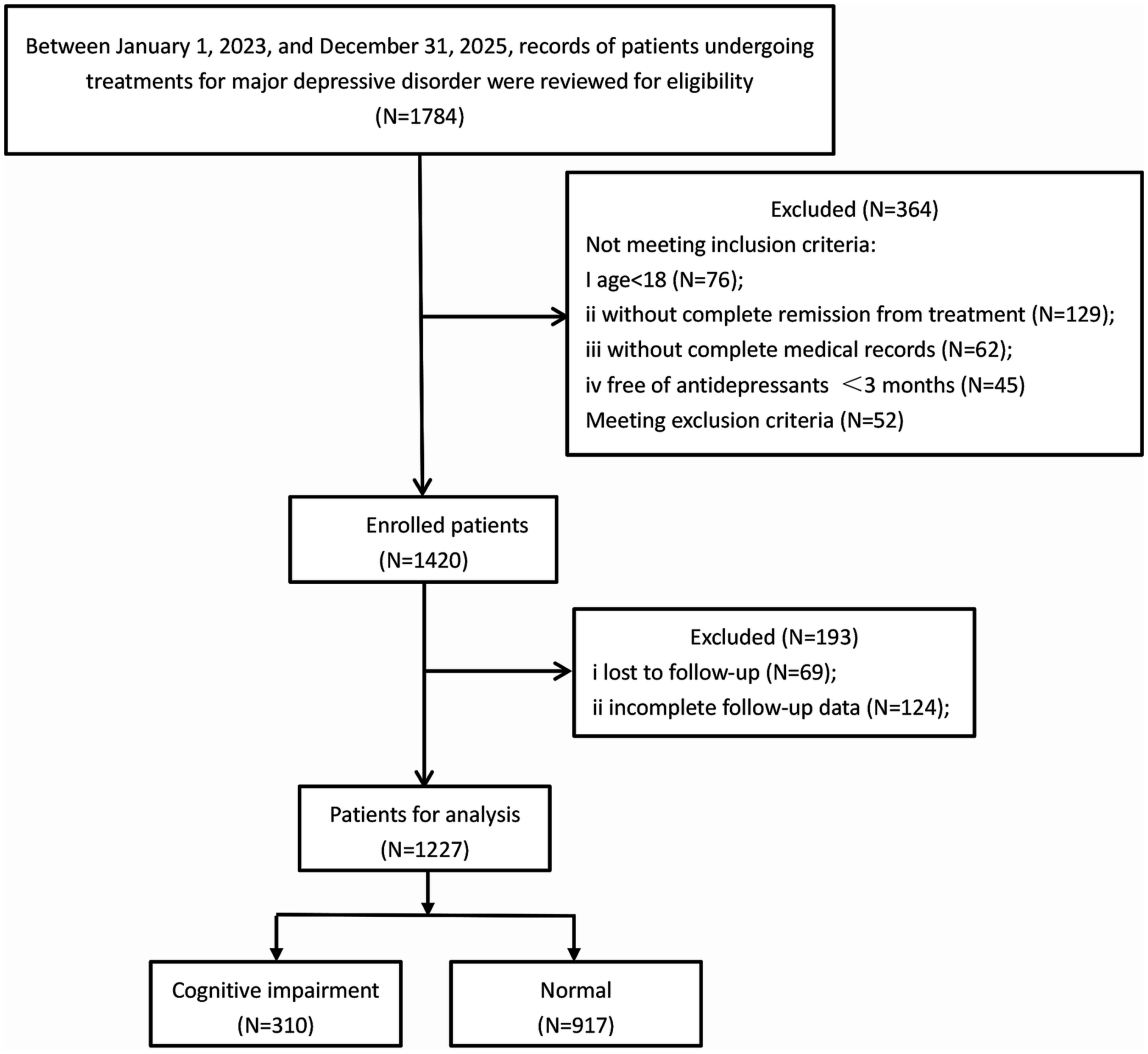
The flowchart of the study cohort.

Figure 1 showed a flowchart of study cohort. A total of 1,227 patients were analyzed, with 25.3% found to have cognitive impairment based on the computerized Testing results from the THINC-it tool. Compared to patients with normal cognitive function, those who experienced cognitive impairment after remitting from MDD had a significantly higher rate of recurrent MDD. In addition, they exhibited higher levels of proinflammatory cytokines, including CRP, IL-6 and TNF-*α*, as well as higher ISI scores during follow-up (all *p* < 0.001). Conversely, MRI scans showed that the standardized volumes of the dorsolateral prefrontal cortex, thalamus, hippocampus and amygdala were significantly lower in patients with cognitive impairment compared to those with normal cognitive function (all *p* < 0.001). Patients with cognitive impairment displayed reduced task-related activity, indicated by significantly lower percentages of BOLD signal changes on fMRI within the interest region of SCN and VAN. In contrast, these patients exhibited increased activity within the DMN, with significantly higher percentages of BOLD signal changes compared to those with normal cognitive function (all *p* < 0.001) (Tables 1, 2).

**Table 1:** Sociodemographic characteristics of patients with MDD from remission.

Variables	Cognitive function		t/*χ*^2^ value	*P*	All patients (*n =* 1,227)
	Impair (*n =* 310)	Normal (*n =* 917)			
Age, years (mean ± SD)	43.74 ± 12.52	44.38 ± 11.40	−0.833	0.405	44.02 ± 12.11
Gender, *n* (%)			0.217	0.646	
Female	167 (53.9%)	480 (52.3%)			647 (52.7%)
Male	143 (46.1%)	437 (47.7%)			580 (47.3%)
BMI, kg/m^2^ (mean ± SD)	22.28 ± 5.23	22.37 ± 5.05	−0.269	0.788	22.31 ± 5.19
Physical activity, kcal·kg^−1^·wk.^−1^ (mean ± SD)	273.87 ± 30.23	272.80 ± 31.57	0.521	0.602	273.07 ± 32.52
Race, *n* (%)			0.019	0.880	
Han Chinese	230 (74.2%)	684 (74.6%)			914 (74.5%)
Minority	80 (25.8%)	233 (25.4%)			313 (25.5%)
Current smokers, *n* (%)	38 (12.3%)	109 (11.9%)	0.030	0.840	147 (12.0%)
Education years, *n* (%)	10.28 ± 3.05	10.14 ± 3.26	0.686	0.493	10.19 ± 3.17
Marital status, *n* (%)			0.392	0.822	
Single	78 (25.2%)	217 (23.7%)			295 (24.0%)
Married	188 (60.6%)	574 (62.6%)			762 (62.1%)
Divorced	44 (14.2%)	126 (13.7%)			170 (13.9%)
Employment status, *n* (%)			0.208	0.901	
Independent	37 (11.9%)	112 (12.2%)			149 (12.1%)
Part-time employment	78 (25.2%)	219 (23.9%)			297 (24.2%)
Full-time employment	195 (62.9%)	586 (63.9%)			781 (63.7%)
Family monthly income, *n* (%)			0.124	0.940	
<5,000￥	82 (26.5%)	235 (25.6%)			371 (25.8%)
5,000–10,000￥	133 (42.9%)	397 (43.3%)			530 (43.2%)
>10,000￥	95 (30.6%)	285 (31.1%)			380 (31.0%)
Sleep medication use, *n* (%)	41 (13.2%)	124 (12.4%)	0.137	0.694	155 (12.6%)

MDD, major depressive disorder; BMI, body mass index; SD, standard deviation.

**Table 2:** Clinical characteristics of patients with MDD from remission.

Variables	Cognitive function		t/z/*χ*^2^ value	*P*	All patients (*n =* 1,227)
	Impair (*n =* 310)	Normal (*n =* 917)			
Family history of depression, *n* (%)	39 (12.6%)	103 (11.2%)	0.412	0.538	142 (11.6%)
Duration from first depressive episode, years (mean ± SD)	4.75 ± 1.24	4.67 ± 1.42	0.884	0.377	4.70 ± 1.39
Number of depressive episodes, *n* (%)			60.528	<0.001	
First episode	194 (62.6%)	767 (83.6%)			961 (78.3%)
Recurrent	116 (37.4%)	150 (16.4%)			266 (21.7%)
History of treatment-resistant depression episode, *n* (%)	42 (13.5%)	118 (12.9%)	0.095	0.758	160 (13.0%)
Number of hospitalizations since first episode (mean ± SD)	2.93 ± 1.72	2.89 ± 1.54	0.384	0.701	2.94 ± 1.67
Patient-reported frequency of suicide ideation (mean ± SD)	2.54 ± 1.30	2.46 ± 1.18	1.005	0.315	2.49 ± 1.23
Duration of current MDD episode, weeks (mean ± SD)	21.85 ± 9.78	22.03 ± 10.02	−0.275	0.783	21.99 ± 9.86
Depression assessment (mean ± SD)					
HAMD-17 score within 48 h after admission	22.39 ± 4.91	21.96 ± 5.77	1.176	0.240	22.18 ± 5.02
HAMD-17 score at the end of treatments	7.32 ± 4.35	7.40 ± 5.13	−0.246	0.806	7.35 ± 4.48
Treatment setting, *n* (%)			0.097	0.778	
Inpatient	209 (67.4%)	627 (68.4%)			836 (68.1%)
Outpatient	101 (32.6%)	290 (31.6%)			391 (31.9%)
Treatment type, *n* (%)			0.359	0.949	
Antidepressant	251 (81.0%)	736 (80.3%)			987 (80.4%)
Antidepressant with antipsychotic	36 (11.6%)	112 (12.2%)			148 (21.1%)
Antidepressant with antipsychotic and benzodiazepine	15 (4.8%)	49 (5.3%)			64 (5.2%)
Antidepressant with electroconvulsive therapy	8 (2.6%)	20 (2.2%)			28 (2.3%)
Antidepressants type, *n* (%)			1.309	0.727	
SSRI	173 (55.8%)	510 (55.6%)			683 (55.7%)
SNRI	61 (19.7%)	159 (17.3%)			220 (17.9%)
VOR	53 (17.1%)	176 (19.2%)			229 (18.7%)
Other	23 (7.4%)	72 (7.9%)			95 (7.7%)
Antidepressant exposure duration, months (mean ± SD)	15.25 ± 3.67	14.98 ± 3.10	1.263	0.207	15.03 ± 3.49
Remission duration, months (mean ± SD)	16.93 ± 6.55	16.58 ± 6.71	0.799	0.425	16.70 ± 6.38
Proinflammatory cytokines (mean ± SD)					
CRP (mg/ml)	3.34 ± 0.59	1.29 ± 0.36	72.601	<0.001	2.65 ± 0.48
IL-6 (pg/ml)	7.76 ± 1.13	5.89 ± 1.32	22.39	<0.001	6.35 ± 1.26
TNF-α(pg/ml)	8.85 ± 1.15	6.74 ± 1.93	18.185	<0.001	7.37 ± 1.82
Brain volumetry on MRI, mm^3^ (mean ± SD)					
Dorsolateral prefrontal cortex	19403.55 ± 322.15	19816.43 ± 367.29	−17.631	<0.001	19683.72 ± 348.91
Thalamus	7391.40 ± 607.25	7814.88 ± 616.53	−10.495	<0.001	7552.64 ± 614.31
Hippocampus	3027.17 ± 220.89	3674.71 ± 391.37	−27.674	<0.001	3293.44 ± 320.19
Amygdala	1106.58 ± 270.45	1686.44 ± 299.82	−30.155	<0.001	1429.13 ± 283.96
Percentage BOLD signal change on fMRI (mean ± SD)					
Default mode network	−0.42 ± 0.15	−0.75 ± 0.12	−39.171	<0.001	−0.58 ± 0.13
Subcortical network	0.59 ± 0.17	0.91 ± 0.13	−34.504	<0.001	0.75 ± 0.15
Ventral attention network	0.45 ± 0.11	0.78 ± 0.14	−37.746	<0.001	0.59 ± 0.12
Common psychiatric comorbidities, *n* (%)					
Generalized anxiety disorder	58 (18.7%)	157 (17.1%)	0.405	0.545	215 (17.5%)
Panic disorder	24 (7.7%)	56 (6.1%)	1.016	0.351	80 (6.5%)
Post-traumatic stress disorder	16 (5.2%)	53 (5.8%)	0.167	0.776	69 (5.6%)
Obsessive-compulsive disorder	19 (6.1%)	43 (4.7%)	1.001	0.368	62 (5.1%)
Antisocial personality disorder	12 (3.9%)	25 (2.7%)	1.038	0.337	37 (3.0%)
Common physical comorbidities, *n* (%)					
Hypertension	81 (26.1%)	235 (25.6%)	0.031	0.881	316 (25.8%)
Diabetes	48 (15.6%)	140 (15.3%)	0.008	0.927	188 (15.3%)
Cardiovascular disease	38 (12.3%)	102 (11.1%)	0.295	0.606	140 (11.4%)
Other	33 (10.6%)	105 (11.5%)	0.150	0.756	138 (11.2%)
Ongoing additional psychotherapy, *n* (%)	65 (21.0%)	186 (20.3%)	0.067	0.796	251 (20.5%)
Disease-related 30-day readmission rates, *n* (%)	12 (3.9%)	42 (4.6%)	0.277	0.749	54 (4.4%)
ISI score at last day of follow-up (mean ± SD)	15.62 ± 3.19	6.28 ± 3.21	44.357	<0.001	9.92 ± 2.75
WHOQOL-BREF score at last day of follow-up (mean ± SD)	64.09 ± 15.62	65.13 ± 16.93	−0.953	0.341	64.93 ± 16.41

MDD, major depressive disorder; HAMD-17, 17-item Hamilton Rating Scale for Depression; HAMA, Hamilton Anxiety Scale; SSRI, selective serotonin reuptake inhibitor; SNRI, serotonin norepinephrine reuptake inhibitors; VOR, vortioxetine; CRP, C-reactive protein; IL-6, interleukin-6; TNF-α, tumor necrosis factor-α; MRI, Magnetic Resonance Images; BOLD, blood oxygen level dependent; fMRI, functional magnetic Resonance Images; ISI, Insomnia Severity Index; WHOQOL-BREF, World Health Organization Quality of Life Brief version; SD, standard deviation.

**Figure 2:**
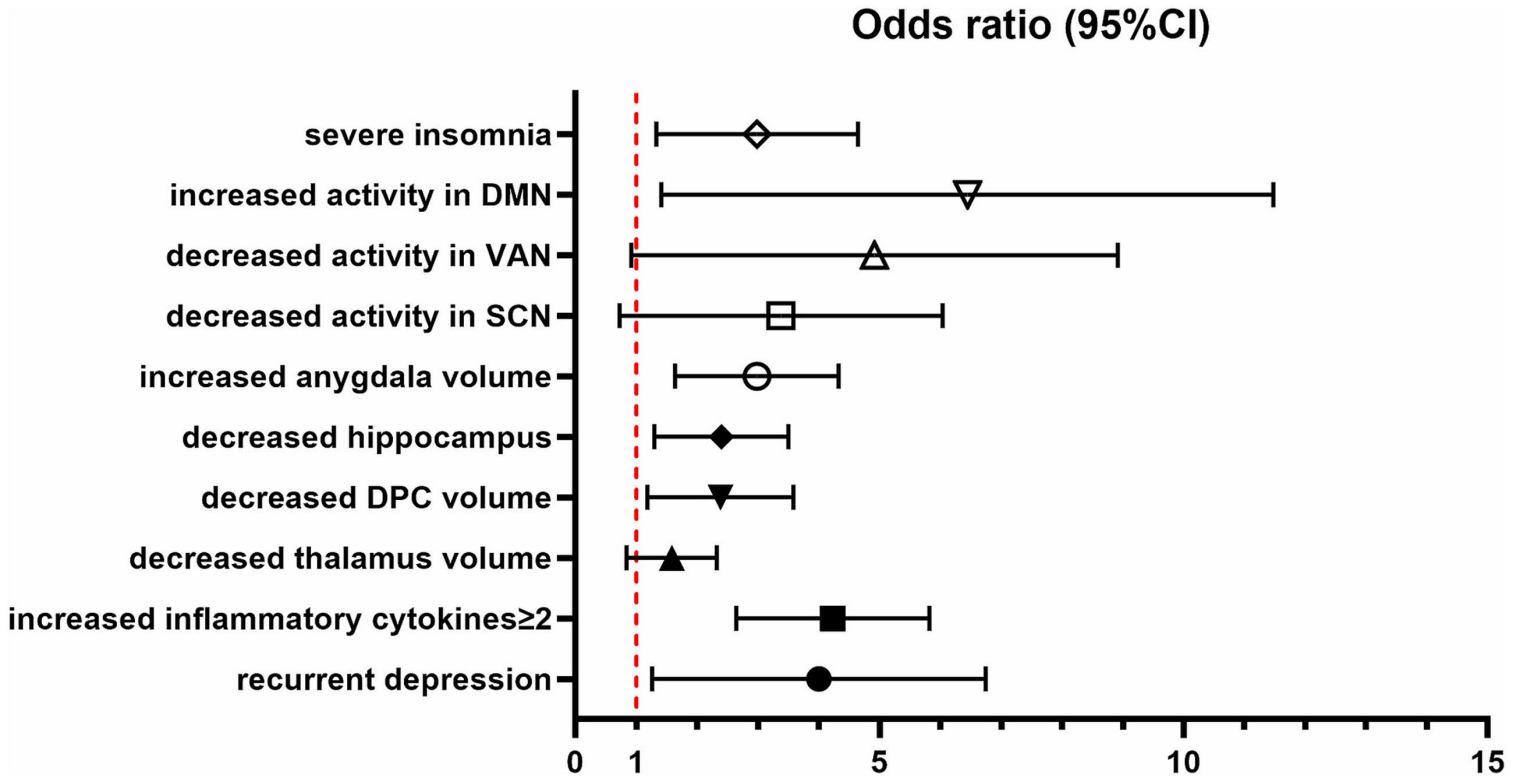
The forest plot summarized the multivariate analyses of risk factors associated with cognitive impairment in patients with MDD following remission. MDD, major depressive disorder; DPC, dorsolateral prefrontal cortex; DMN, default mode network; SCN, subcortical network; VAN, ventral attention network.

According to the Pearson correlation analysis, there is a significant correlation among there inflammatory cytokines. Consequently, patients were categorized into two subgroups on the basis of the number of elevated inflammatory cytokines. Additionally, no multicollinearity was detected among the variables in logistic regression. All baseline variables were entered into the multiple logistic regression model using a likelihood ratio stepwise method. As shown in Table 3 and Figure 2, recurrent MDD (OR = 3.377, 95% CI: 1.629–7.003), number of increased inflammatory cytokines≥2 (OR = 4.032, 95% CI: 2.751–5.909), decreased dorsolateral prefrontal cortex volume (OR = 2.184, 95% CI: 1.298–3.676), decreased hippocampus volume (OR = 2.232, 95% CI: 1.393–3.576), increased amygdala volume (OR = 2.782, 95% CI: 1.751–4.420), decreased percentage of BOLD signal change for activity in SCN (OR = 2.689, 95% CI: 1.145–6.313) and VAN (OR = 3.834, 95% CI: 1.573–9.345), as well as increased percentage of BOLD signal change for activity in DMN (OR = 5.136, 95% CI: 2.198–12.005) and ISI scores ≥22 points (OR = 2.679, 95% CI: 1.502–4.779) were independent risk factors for experiencing cognitive impairment in patients within remission from MDD. The individual PI value was calculated using the following formula:

**Table 3:** Multiple logistic regression analysis for predicting cognitive impair in patients with MDD after remission.

Variables	Regression coefficient			Adjusted odds ratio			*P*
	B	SE	Wald *χ*^2^	Exp (B)	95% CI		
					Lower	Upper	
Number of depressive episodes							
First episode							
Recurrent	1.217	0.372	10.691	3.377	1.628	7.003	<0.001
Number of increased inflammatory cytokines							
Normal							
≥2	1.394	0.195	51.095	4.032	2.751	5.909	<0.001
Brain volumetry on MRI			22.768				<0.001
Normal							
Decreased thalamus volume	0.388	0.244	2.539	1.457	0.915	2.378	0.111
Decreased dorsolateral prefrontal cortex volume	0.781	0.266	8.654	2.184	1.298	3.676	0.003
Decreased hippocampus volume	0.803	0.240	11.149	2.232	1.393	3.576	<0.001
Increased amygdala volume	1.023	0.236	18.774	2.782	1.751	4.420	<0.001
Percentage BOLD signal change for activity on fMRI			20.814				<0.001
Normal							
Subcortical network < mean value of 0.75	0.912	0.433	4.429	2.689	1.145	6.313	0.023
Ventral attention network < mean value of 0.59	1.344	0.455	8.737	3.834	1.573	9.345	0.003
Default mode network > mean value of −0.58	1.636	0.433	14.271	5.136	2.198	12.005	<0.001
Severe insomnia with ISI score≥22 points	0.985	0.295	11.132	2.679	1.502	4.779	<0.001
Constant	−3.429	0.461	55.393	0.032			<0.001

MDD, major depressive disorder; MRI, Magnetic Resonance Images; BOLD, blood oxygen level dependent; fMRI, functional magnetic Resonance Images; ISI, Insomnia Severity Index.

PI = 1.127 × recurrent MDD + 1.394 × Number of increased inflammatory cytokines ≥2 + 0.781 × decreased dorsolateral prefrontal cortex volume+0.803 × decreased hippocampus volume+1.023 × increased amygdala volume+0.912 × percentage of BOLD change for activity in SCN < mean value+1.344 × percentage of BOLD change for activity in VAN < mean value+1.636 × percentage of BOLD change for activity in DMN > mean value+0.958 × ISI score≥22–3.429.

**Figure 3:**
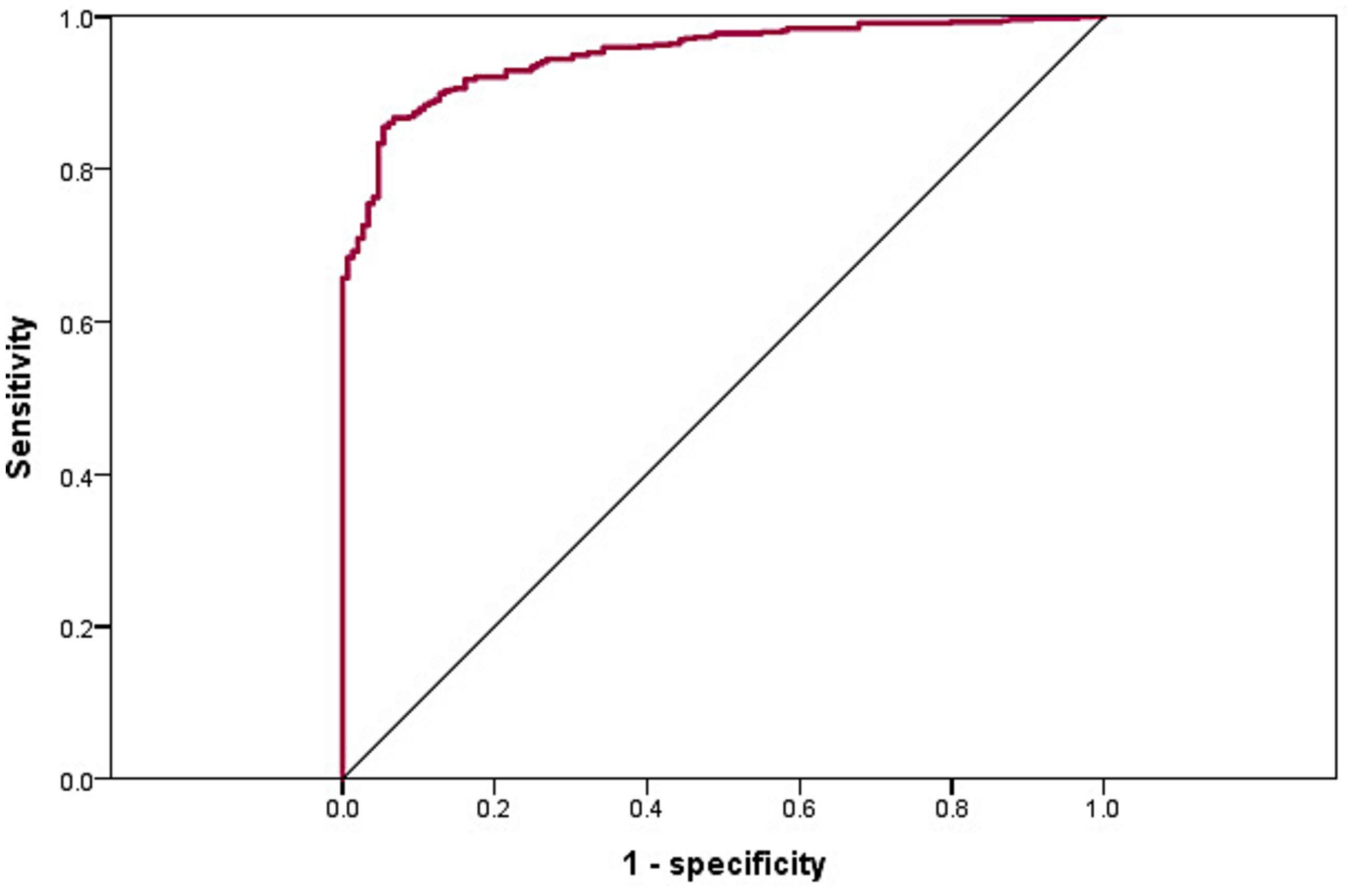
ROC curves for the prediction model. The AUC was 0.902 (95% CI: 0.875–0.930) with the Youden index value of 0.740, a sensitivity of 90.3% and a specificity of 83.6%. ROC, Receiver operating characteristic; AUC, area under the curve.

According to ROC analysis, the area under the curve (AUC) was 0.902 (95% CI: 0.875–0.930) (*p* < 0.001). The optimum cutoff value was 0.148 based on the Youden index value = 0.740, yielding a sensitivity of 90.3% and a specificity of 83.6% (Figure 3).

## Discussion

Our results showed that recurrent MDD, increased inflammatory cytokines, decreased volumes in dorsolateral prefrontal cortex and hippocampus, increased volumes in amygdala, decreased activity in SCN and VAN, increased activity in DMN, as well as severe insomnia were independent risk factors for cognitive impairment in MDD patients following remission. It also proposed a potentially effective method for promptly identifying patients who may be at risk.

According to a prior study investigating cognitive function in depressive disorders, the severity of depressive symptoms was similar between first-episode depression (ED-I) and recurrent depressive disorder (rDD). However, cognitive function in terms of processing speed, learning, visual and verbal memory, working memory, executive functions and verbal fluency was significantly better in the ED-I group compared to the rDD group (Talarowska et al., 2015). A recent systematic review of cognitive impairment in both the acute and remitted states of MDD also revealed that a greater number of MDD episodes was significantly associated with cognitive decline (Kriesche et al., 2023). Consistent with earlier evidence, our results showed that patients with a recurrent episode of MDD were more likely to experience cognitive impairment after achieving remission from MDD than those with a first depressive episode.

In the past decades, increasing evidence indicated that the immune-inflammatory processes played a significant role in the pathogenesis of MDD. During acute phase, inflammatory agents infiltrated the central nervous system, leading to various abnormal behavioral, cognitive and emotional responses in patients with MDD (Xi et al., 2024). Recently, Yuan et al. (2026) investigated the cognitive performance in patients with MDD using the Montreal cognitive assessment (MoCA). Their findings indicated that serum levels of IL-6, TNF-*α*, and CRP were negatively correlated with MoCA scores (r = −0.376 to −0.401, *p* < 0.001), suggesting that higher levels of inflammatory cytokines were significantly associated with poor cognitive function in MDD patients. Our results supported these findings, demonstrating that patients with two or more increased inflammatory cytokines were more likely to exhibit cognitive impairment after remission from MDD, compared to those with normal inflammatory levels.

According to a review of cognitive impairment in depression, dysfunctions in neural networks and brain morphometry in specific areas were responsible for the persistence of cognitive impairment even after symptomatic remission (Czerwinska and Pawlowski, 2020). The activation of the hypothalamic–pituitary–adrenal (HPA) axis caused oxidative stress, resulting in cellular damage and structural atrophy in hippocampus, which significantly influenced emotion processing in cognitive function (Lei et al., 2025). In contrast, chronic stress in MDD also contributed to dendritic remodeling and volume enlargement in amygdala, resulting in difficulties with emotion generation and regulation, as well as challenges in learning and memory management (Li et al., 2025). Based on the results from a meta-analysis of MRI studies, patients with MDD and mild cognitive impairment showed common volume reduction in several brain regions such as prefrontal cortex, thalamus and hippocampus (Zackova et al., 2021). Our findings indicated that alterations in brain volume negatively affected cognitive function, characterized by a reduction in the volume of dorsolateral prefrontal cortex and hippocampus volume, alongside an increase in the volume of amygdala.

In patients with MDD, both attention and executive function were the most impaired cognitive domains. A previous review indicated that attention deficits were related to dysfunctions in large-scale brain networks such as frontoparietal network, dorsal attention network and ventral attention network. Meanwhile, executive dysfunction was associated with abnormal activity in the dorsal prefrontal cortex (Li et al., 2024). These findings were further supported by a recent large-scale, cross-sectional fMRI study, in which all participants underwent scanning during a verbal working memory N-back task. Compared to healthy controls, patients with MDD showed increased activity in the DMN and decreased activity in the prefrontal cortex and inferior frontal gyrus, and temporal-occipital areas (Damgaard et al., 2025). Similarly, our findings suggested that patients exhibiting reduced activity in the SCN and VAN, along with increased activity in the DMN, as measured by BOLD fMRI, were more likely to exhibit cognitive impairment after remitting from MDD compared to those with normal activity levels.

Regarding other clinical factors, sleep problems occurred in approximately 85% of patients with a major depressive episode, and continued in 39% of cases in remission (Fortier-Brochu and Morin, 2014). A recent study reported that poor sleep efficiency directly affected patients’ cognitive function, leading to poor executive function (*p* = 0.004), lower processing speed (*p* = 0.047) and memory impairments (*p* < 0.001). Furthermore, sleep difficulties served as a mediator between psychic anxiety (*β* = −0.013) and declines in both executive function and memory (*β* = −0.149), resulting in a significant impairment in cognitive function (Wang et al., 2024). Aligning with their results, we found that patients who reported severe insomnia during follow-up were more likely to have cognitive impairment with remission from MDD, compared to those with normal sleep quality.

The study had several limitations. Firstly, the retrospective data in a single-center might produce possible selection bias and reduced predictive generalizability. Secondly, although high ROC performance was impressive, the absence of internal and external validations raised the possibility of overfitting, limiting the generalizability of our findings. Thirdly, the HAMD-17, ISI and WHOQoL-BREF questionnaires assessed overlapping psychometric aspects. Fourthly, medication confounding insufficiently addressed. Fifthly, patients who were uncomfortable with technology might have difficulty in completing computerized assessments. Lastly, lack of long-term follow-up might affect interpretation of cognitive outcomes. Therefore, future research should involve a well-designed randomized controlled study to verify our findings.

## Conclusion

In conclusion, our research reported risk factors associated with cognitive impairment in patients who achieved remission from MDD. Furthermore, we developed a straightforward framework to help healthcare providers in identifying patients at higher risk of experiencing cognitive impairment. This tool was suggested for use in evaluating risk factors during the treatment of patients with MDD to proceed early preventive interventions for this issue.

## Data Availability

The data are available from the corresponding author upon reasonable request.

## Footnotes

1
http://atlas.brainnetome.org

## Glossary

MDD: major depression disorder
THINC-it: THINC-integrated tool
EMRs: Electronic Medical Record
HAMD-17: 17-item Hamilton Rating Scale for Depression
CRP: C-reactive protein
IL-6: interleukin-6
TNF-*α*: tumor necrosis factor-α
MRI: magnetic resonance imaging
fMRI: functional magnetic resonance imaging
BOLD: Blood oxygen level dependent
MID: monetary incentive delay
DMN: default mode network
SCN: subcortical network
VAN: ventral attention network
PDQ-5-D: Perceived Deficits Questionnaire-5-item
SPO: Spotter
SC: Symbol Check
CB: Code Breaker
TRA: Trails
ISI: Insomnia Severity Index
WHOQOL-BREF: World Health Organization Quality of Life Brief version
SD: standard deviation
PI: prognostic index
ROC: receiver-operating characteristic curve
AUC: area under the curve.

## References

[ref1] Bains N. Abdijadid S. (2025). Major Depressive Disorder. Treasure Island, FL: Statpearls. 32644504

[ref2] Bastien C. H. Vallieres A. Morin C. M. (2001). Validation of the insomnia severity index as an outcome measure for insomnia research. Sleep Med. 2, 297–307. doi: 10.1016/s1389-9457(00)00065-4, 11438246

[ref3] Casamali F. F. C. Schuch F. B. Scortegagna S. A. Legnani E. De Marchi A. C. B. (2019). Accordance and reproducibility of the electronic version of the Whoqol-Bref and Whoqol-old questionnaires. Exp. Gerontol. 125:110683. doi: 10.1016/j.exger.2019.110683, 31398444

[ref4] Cokmus F. P. Ozkan H. M. Suculluoglu-Dikci D. Suculluoglu Dikici D. Ascibasi K. Alci D. . (2021). The assessment of cognitive dysfunction in major depressive disorder: a 16-week prospective case-control study. Psychiatry Clin. Psychopharmacol. 31, 25–33. doi: 10.5152/pcp.2021.20148, 39619350 PMC11605319

[ref5] Czerwinska A. Pawlowski T. (2020). Cognitive dysfunctions in depression - significance, description and treatment prospects. Psychiatr. Pol. 54, 453–466. doi: 10.12740/PP/OnlineFirst/105415, 33038880

[ref6] Damgaard V. Schandorff J. M. Macoveanu J. . (2025). Network-wide aberrancies in neuronal activity during working memory in a large cohort of patients with mood disorders: associations with cognitive impairment and functional disability. Mol. Psychiatry 30, 4836–4844. doi: 10.1038/s41380-025-03078-x, 40527901 PMC12436155

[ref7] Douglas K. M. Milanovic M. Porter R. J. Bowie C. R. (2020). Clinical and methodological considerations for psychological treatment of cognitive impairment in major depressive disorder. BJPsych Open 6:e67. doi: 10.1192/bjo.2020.53, 32594951 PMC7345587

[ref8] First M. B. (2013). Diagnostic and statistical manual of mental disorders, 5th edition, and clinical utility. J. Nerv. Ment. Dis. 201, 727–729. doi: 10.1097/NMD.0b013e3182a2168a, 23995026

[ref9] Fortier-Brochu E. Morin C. M. (2014). Cognitive impairment in individuals with insomnia: clinical significance and correlates. Sleep 37, 1787–1798. doi: 10.5665/sleep.4172, 25364074 PMC4196062

[ref10] Hashemzadeh I. Marquez-Arrico J. E. Hashemzadeh K. Navarro J. F. Adan A. (2021). Circadian functioning and quality of life in substance use disorder patients with and without comorbid major depressive disorder. Front. Psych. 12:750500. doi: 10.3389/fpsyt.2021.750500, 34777054 PMC8586202

[ref11] Hou Y. Yao S. Hu S. . (2020). Psychometric properties of the Chinese version of the Thinc-it tool for cognitive symptoms in patients with major depressive disorder. J. Affect. Disord. 273, 586–591. doi: 10.1016/j.jad.2020.03.146, 32560957

[ref12] Knight M. J. Baune B. T. (2018). Cognitive dysfunction in major depressive disorder. Curr. Opin. Psychiatry 31, 26–31. doi: 10.1097/YCO.0000000000000378, 29076892

[ref13] Kriesche D. Woll C. F. J. Tschentscher N. Engel R. R. Karch S. (2023). Neurocognitive deficits in depression: a systematic review of cognitive impairment in the acute and remitted state. Eur. Arch. Psychiatry Clin. Neurosci. 273, 1105–1128. doi: 10.1007/s00406-022-01479-5, 36048295 PMC10359405

[ref14] Kundu P. Inati S. J. Evans J. W. Luh W. M. Bandettini P. A. (2012). Differentiating Bold and non-Bold signals in Fmri time series using multi-Echo epi. NeuroImage 60, 1759–1770. doi: 10.1016/j.neuroimage.2011.12.028, 22209809 PMC3350785

[ref15] Lam R. W. Kennedy S. H. McLntyre R. S. Khullar A. (2014). Cognitive dysfunction in major depressive disorder: effects on psychosocial functioning and implications for treatment. Can. J. Psychiatr. 59, 649–654. doi: 10.1177/070674371405901206, 25702365 PMC4304584

[ref16] Lei A. A. Phang V. W. X. Lee Y. Z. Kow A. S. F. Tham C. L. Ho Y.-C. . (2025). Chronic stress-associated depressive disorders: the impact of Hpa axis dysregulation and neuroinflammation on the hippocampus-a mini review. Int. J. Mol. Sci. 26:2940. doi: 10.3390/ijms26072940, 40243556 PMC11988747

[ref17] Li B. Zhang C. Chen W. Xie G. Liang J. (2025). Amygdala volume abnormalities and cognitive impairment in major depressive disorder and bipolar disorder ii. BMC Psychiatry 25:839. doi: 10.1186/s12888-025-07313-1, 40877858 PMC12395685

[ref18] Li Y. T. Zhang C. Han J. C. . (2024). Neuroimaging features of cognitive impairments in schizophrenia and major depressive disorder. Ther Adv Psychopharmacol 14:20451253241243290. doi: 10.1177/20451253241243290, 38708374 PMC11070126

[ref19] Malhi G. S. Mann J. J. (2018). Depression. Lancet 392, 2299–2312. doi: 10.1016/S0140-6736(18)31948-2, 30396512

[ref20] McIntyre R. S. Best M. W. Bowie C. R. . (2017). The Thinc-integrated tool (Thinc-it) screening assessment for cognitive dysfunction: validation in patients with major depressive disorder. J. Clin. Psychiatry 78, 873–881. doi: 10.4088/JCP.16m11329, 28858441

[ref21] Miguel N. Marquez-Arrico J. E. Jodar M. Navarro J. F. Adan A. (2023). Neuropsychological functioning of patients with major depression or bipolar disorder comorbid to substance use disorders: a systematic review. Eur. Neuropsychopharmacol. 75, 41–58. doi: 10.1016/j.euroneuro.2023.06.006, 37453267

[ref22] Nierenberg A. A. DeCecco L. M. (2001). Definitions of antidepressant treatment response, remission, nonresponse, partial response, and other relevant outcomes: a focus on treatment-resistant depression. J. Clin. Psychiatry 62, 5–9. 11480882

[ref23] Pan Z. Grovu R. C. Cha D. S. Carmona N. E. Subramaniapillai M. Shekotikhina M. . (2017). Pharmacological treatment of cognitive symptoms in major depressive disorder. CNS Neurol. Disord. Drug Targets 16, 891–899. doi: 10.2174/1871527316666170919115100, 28933261

[ref24] Talarowska M. Zajaczkowska M. Galecki P. (2015). Cognitive functions in first-episode depression and recurrent depressive disorder. Psychiatr. Danub. 27, 38–43. 25751430

[ref25] Vandenbroucke J. P. von Elm E. Altman D. G. Gøtzsche P. C. Mulrow C. D. Pocock S. J. . (2014). Strengthening the reporting of observational studies in epidemiology (Strobe): explanation and elaboration. Int. J. Surg. 12, 1500–1524. doi: 10.1016/j.ijsu.2014.07.014, 25046751

[ref26] Wang L. Li L. Zhang R. Zhao J. Wei M. Nie Z. (2025). Comparison of disease burden of major depressive disorder in China and globally from 1991 to 2021. J. Affect. Disord. 390:119816. doi: 10.1016/j.jad.2025.119816, 40617487

[ref27] Wang M. Chen W. T. Wang H. T. Liu B. S. Ju Y. M. Dong Q. L. . (2024). Sleep disturbances and psychomotor retardation in the prediction of cognitive impairments in patients with major depressive disorder. World J Psychiatry 14, 1474–1483. doi: 10.5498/wjp.v14.i10.1474, 39474372 PMC11514569

[ref28] Xi Y. Q. Wang Z. Q. Li G. J. Hao Z. Q. Nie J. H. Li J. X. . (2024). Association of Inflammation Cytokines with cognitive function in First-episode major depressive disorder. Front. Psych. 15:1473418. doi: 10.3389/fpsyt.2024.1473418, 39911552 PMC11794534

[ref29] Yuan Q. Wang L. Zhu X. Wu Z. He S. Zhang H. . (2026). Metabolic dysfunction and cognitive impairment in patients with depression: the role of the tyg index and peripheral inflammation. J. Affect. Disord. 397:121026. doi: 10.1016/j.jad.2025.121026, 41443310

[ref30] Zackova L. Jani M. Brazdil M. Nikolova Y. S. Mareckova K. (2021). Cognitive impairment and depression: Meta-analysis of structural magnetic resonance imaging studies. Neuroimage Clin 32:102830. doi: 10.1016/j.nicl.2021.102830, 34560530 PMC8473769

[ref31] Zhang S. Zhou J. Cui J. Zhang Z. Liu R. Feng Y. . (2023). Effects of 12-week escitalopram treatment on resting-state functional connectivity of large-scale brain networks in major depressive disorder. Hum. Brain Mapp. 44, 2572–2584. doi: 10.1002/hbm.26231, 36773284 PMC10028676

[ref32] Zhu N. Zhang Q. Huang J. Tong J. Gong H. F. Zhu M. H. . (2025). Using the Thinc-integrated tool to compare the characteristics of cognitive dysfunction in patients with unipolar and bipolar depression. World J Psychiatry 15:99408. doi: 10.5498/wjp.v15.i3.99408, 40110017 PMC11886334

